# Genomic risk prediction of type 2 diabetes in people living with and without HIV

**DOI:** 10.1038/s41598-025-31471-7

**Published:** 2026-01-22

**Authors:** Nicole D. Armstrong, Vinodh Srinivasasainagendra, Lavanya Pilla, Radhika Gangaraju, Peter W. Hunt, Robin M. Nance, Heidi M. Crane, Inga Peter, Sonya L. Heath, Greer A. Burkholder, Richard D. Moore, Jeffrey M. Jacobson, Edward R. Cachay, Thibaut Davy-Mendez, Hirotaka Iwaki, Lana Sargent, Hemant K. Tiwari, Marguerite R. Irvin

**Affiliations:** 1 Department of Epidemiology, University of Alabama at Birmingham, Birmingham, AL USA; 2 Department of Biostatistics, University of Alabama at Birmingham, Birmingham, AL USA; 3 Department of Medicine, University of Alabama at Birmingham, Birmingham, AL USA; 4 Division of Experimental Medicine, University of California, San Francisco, San Francisco, CA USA; 5 Department of Medicine, University of Washington, Seattle, WA USA; 6 Department of Genetics and Genomic Sciences, Icahn School of Medicine at Mount Sinai, New York, NY USA; 7 Department of Medicine, Johns Hopkins University, Baltimore, MD USA; 8 Department of Medicine, Case Western Reserve University, Cleveland, OH USA; 9 Department of Medicine, Division of Infectious Diseases, Mayo Clinic, Scottsdale, AZ USA; 10 School of Medicine, University of North Carolina at Chapel Hill, Chapel Hill, NC USA; 11 Gillings School of Public Health, University of North Carolina at Chapel Hill, Chapel Hill, NC USA; 12 Center for Alzheimer’s and Related Dementias, National Institute on Aging, Bethesda, MD USA; 13 DataTecnica LLC, Washington, DC USA; 14 School of Nursing, Virginia Commonwealth University, Richmond, VA USA

**Keywords:** Polygenic risk score, Precision medicine, Type 2 diabetes, HIV, Biomarkers, Diseases, Endocrinology, Genetics, Medical research, Risk factors

## Abstract

Type 2 diabetes (T2D) risk prediction remains a challenge, particularly in underrepresented populations, including people living with HIV (PWH) and those of non-European ancestry. We evaluated the performance of two metaPRS (polygenic risk score) models, integrating genetic markers related to inflammation and lipid metabolism, in predicting T2D risk across ancestry groups (African and European), with and without HIV. The metaPRS were generated in a subset from the Reasons for Geographic and Racial Differences in Stroke (REGARDS) study (6,034 Black; 11,972 White) and validated in 7,580 (4,120 Black; 3,460 White) PWH from the Centers for AIDS Research of Integrated Clinical Systems (CNICS), as well as an additional 4,152 (2,586 Black; 1,566 White) seronegative participants from REGARDS. Incorporating the metaPRS into models provided non-significant improvements in T2D risk prediction compared to single-trait T2D PRS and clinical risk factors. Performance was similar in PWH and in people without HIV, suggesting that these general population-derived genetic scores are transferable to PWH. Future studies should focus on refining PRS models in diverse populations and exploring genetic factors specific to PWH regarding T2D risk.

**Supplementary Information:**

The online version contains supplementary material available at 10.1038/s41598-025-31471-7.

## Introduction

Type 2 diabetes (T2D) is a complex and heterogeneous condition characterized by glucose dysregulation and insulin resistance, and is the most common form of diabetes, comprising > 90% of individuals with diabetes in the United States^1^. Chronic hyperglycemia contributes to long-term complications, including dysfunction and damage to the kidneys, heart, and blood vessels, making diabetes a major risk factor for cardiovascular diseases^2^. Despite advances in treatment and management, the growing prevalence of T2D^3^ underscores the need for improved risk prediction and early intervention strategies tailored to individual patients.

T2D has a strong genetic component in the general population, with heritability estimates from twin and family studies ranging from 30% to 70%^4–7^and array-based analyses ranging from 18% to 34%^8^. Genome-wide association studies (GWAS) have identified over 500 genetic variants associated with T2D^9–11^, highlighting its complex polygenic architecture. Polygenic risk scores (PRS), which aggregate the effects of multiple genetic variants associated with disease risk, have emerged as promising tools for predicting future T2D and improving early diagnosis and prevention efforts^12^. However, PRS have primarily been developed using populations of European ancestry, which may limit their predictive accuracy and generalization to other populations^13,14^ or those with coexisting chronic conditions.

The precision medicine paradigm seeks to tailor disease prevention, diagnosis, and treatment to individual patients by leveraging genetic, environmental, and clinical data^15^. In multi-factorial diseases, such as T2D, integrating genetic biomarkers (e.g., PRS) into clinical practice offers the potential to identify high-risk individuals and customize interventions^16^. Recent advances in genetic risk modeling have enhanced PRS construction by combining multiple PRS or sets of summary statistics into a meta-score (metaPRS)^17^. MetaPRS approaches have shown improvement in risk prediction over single-trait PRS in other complex diseases, such as coronary artery disease (CAD)^18^ and ischemic stroke^17^, by capturing a more comprehensive genetic risk profile. We hypothesize that incorporating genetic markers related to inflammation and lipid metabolism into metaPRS models may improve T2D prediction, particularly in populations with HIV where these inflammatory biological pathways play a critical role.

People living with HIV (PWH) face an elevated risk of T2D compared to the general population^19,20^, driven by a complex interplay of traditional and HIV-specific risk factors, including disruptions in lipid metabolism^20,21^, chronic systemic inflammation, and insulin resistance^20^. Some antiretroviral therapy (ART) agents have been linked to metabolic side effects, including weight gain, dyslipidemia, and insulin resistance^22^, further compounding the risk of T2D in PWH. Genetic variation in inflammatory and lipid metabolism pathways may contribute to this heightened risk, but our understanding of the role of these genetic markers in T2D prediction among PWH remains limited.

In the current study, we evaluated the utility of metaPRS models for predicting prevalent T2D in populations with and without HIV across two ancestry groups (African and European), utilizing genetic and phenotypic data from 22,158 participants in the Reasons for Geographic and Racial Differences in Stroke (REGARDS) study and 7,580 participants from the Centers for AIDS Research of Integrated Clinical Systems (CNICS) cohort. Specifically, we examined whether a general population T2D score translates to an HIV population and whether the inclusion of genetic markers related to inflammation or lipid metabolism improved the predictive accuracy of T2D in PWH compared to seronegative individuals. This research underscores the potential of genetic risk models, such as metaPRS, as precision medicine tools to enhance risk prediction and inform targeted interventions.

## Methods

### Study design and participants

#### Reasons for geographic and racial differences in stroke (REGARDS) study

The REGARDS study is a national, longitudinal study of incident stroke and associated risk factors, enrolling over 30,000 self-identified Black and White adults aged 45 years or older from all 48 contiguous US states and the District of Columbia^23^. Participants completed a computer-assisted telephone interview (CATI) and an in-home visit where blood and urine were collected, and a medication inventory was taken. Participants are contacted at six-month intervals to obtain information regarding incident stroke or secondary outcomes. Institutional review boards approved this study at all participating institutions, and all participants provided written informed consent.

Genotyping in REGARDS was conducted in two independent batches. The first batch included 10,788 participants (84% Black), genotyped on the Illumina Infinium AMR/AFR (MEGA) BeadChip array. Quality control procedures on the sample and variant level have been previously described^24^. Briefly, variants were excluded if they were located on sex chromosomes, had ambiguous strands, were multi-allelic, violated Hardy Weinberg Equilibrium (HWE, *p* < 1.00e-12), and/or had a missing rate > 10%. Individuals were excluded based on sex discrepancies (genotyped versus self-reported), internal duplicates, or HapMap controls. Imputation was performed using the Trans-omics for Precision Medicine (TOPMed) release 2 (Freeze 8) reference panel^25^. Principal components (PCs) were generated using EIGENSTRAT^26^ to account for population substructure and as a measure of genetic similarity.

A second, independent batch included 12,118 participants of European ancestry genotyped on the Illumina Infinium Global Diversity Array-9 (GDA) with custom content targeting neurodegenerative disease-focused variants^27^. Variants were excluded if they were located on sex chromosomes, had ambiguous strands, were multi-allelic, were indels, violated Hardy Weinberg Equilibrium (HWE, *p* < 1.00e-05), and/or had a missing rate > 5%. Individuals were excluded based on sex discrepancies (genotyped versus self-reported), internal duplicates, or those with < 98% call rates. Imputation was performed using the Trans-omics for Precision Medicine (TOPMed) release 3 reference panel^25^. PCs were generated using EIGENSTRAT.

T2D in REGARDS was defined based on fasting glucose ≥ 126 mg/dL (7 mmol/L), non-fasting glucose ≥ 200 mg/dL (11.1 mmol/L), or the use of diabetes medications (e.g., oral hypoglycemic pills or insulin)^8^. Age, sex, and race were self-reported at baseline. Smoking history was obtained during the CATI, at which participants were asked if they currently smoke cigarettes, if they did in the past, or if they never smoked. For the current study, cigarette smoking was evaluated as current smoking versus past/never smoking. Height and weight were measured during the in-home examination, and body mass index (BMI) was calculated as a continuous variable (kg/m^2^). Systolic blood pressure (SBP) was measured two times following a standardized protocol using an aneroid sphygmomanometer after participants had rested for five minutes^23^. The mean of the two measurements was used to define SBP^28^.

The final analytical sample consisted of 8,620 Black and 13,538 White REGARDS participants passing quality control parameters, with complete genotyping, outcome, and covariate data. For analysis in Black REGARDS participants, the dataset was randomly divided into training (70%, *n* = 6,034) and validation (30%, *n* = 2,586) subsets. In White REGARDS participants, two unique subsets of individuals, based on genotyping platform, were used for training (*n* = 11,972) and validation (*n* = 1,566).

#### The centers for AIDS research of integrated clinical systems (CNICS) cohort

CNICS is a well-characterized longitudinal observation cohort of PWH engaged in clinical care after 1/1/1995 at ten sites across the United States. Full details on CNICS have been previously published^29,30^; however in brief, the CNICS data repository integrates comprehensive clinical data from sites from both outpatient and inpatient visits, including demographic, clinical, and laboratory data, medications, and clinical history^29^. In addition, CNICS has patient reported outcomes (PROs) initiated as part of care in ~ 2009 (date varies by site) including symptoms and risk behaviors such as smoking. CNICS also has biospecimen collection across sites which enables an ongoing genetics project of PWH with multiple racial/ethnic backgrounds.

In CNICS, the methods for genotyping and imputation have been detailed elsewhere^31,32^. Briefly, DNA was extracted from peripheral blood mononuclear cells or buffy coats. Genotyped data was generated using the Illumina Infinium Multi-Ethnic Global Array, the MEGA expanded array, and the Infinium Multi-Ethnic Global-8 Kit. Quality control (QC) for genotyping was conducted before imputation, using PLINK v1.9^33^, and excluded variants with call rates < 95%, MAF < 1%, and deviations from HWE (*p* < 1E-05). Samples with call rates < 90%, sex discrepancies, and pairwise identity-by-descent (pi-hat > 0.9) were also excluded. Ethnicity was inferred using GRAF-pop software^34^, as previously described^32^. Imputation was performed separately for each ancestral group using the TOPMed reference panel, and variants with an imputation quality score were retained. Harmonization across arrays was performed post-imputation. We restricted the analysis to common variants between platforms and used the McCarthy Group Tools^35^ to check for strand flips. The top 10 PCs were calculated using EIGNESTRAT, were ancestry-specific, and were used in subsequent analysis, as well as in harmonization. We tested all variants for associations with the platforms (e.g., MEGA = 1, MEGAex = 0) as a dichotomous outcome, adjusting for the 10 PCs. Variants with *p* < 5E-08 when comparing differences between platforms were removed. This process was repeated to merge all three platforms^36^.

PWH from eight CNICS sites with the relevant data were included in the current study if they had genome-wide genotype data and completed one or more PRO assessments prior to July 2023 (data end date). The T2D definition in CNICS differed slightly from that used in REGARDS. T2D in CNICS was defined as (1) hemoglobin A1c *≥* 6.5% or (2) use of a diabetes-specific medication such as insulin or (3) use of a diabetes-related medication frequently but not exclusively used to treat diabetes (e.g., biguanides) and having a diagnosis of diabetes mellitus^37^. BMI was calculated from height and weight as a continuous variable (kg/m^2^) at initial visit. SBP measures were averaged across all visits. Tobacco use was captured using the CNICS clinical assessment of patient-reported outcomes. CNICS participants self-reported a history of never, former, or current smoking and answered items regarding duration and quantity. We analyzed the cigarette smoking phenotype as current versus non-current and PWH were classified as ART-naïve or ART-experienced. The final analytical sample consisted of 4,120 Black and 3,460 White CNICS participants passing quality control parameters, with complete genotyping, outcome, and covariate data.

This secondary data analysis was approved by the University of Alabama at Birmingham Institutional Review Board (IRB-300010485). All methods were performed in accordance with the relevant guidelines and regulations, including those outlined by the UAB IRB and the Declaration of Helsinki. All REGARDS and CNICS participants provided informed consent.

#### Generation of metaprs

The REGARDS training set was used to construct the metaPRS and was excluded from any further analysis. We applied nine PRS from the Polygenic Score Catalog^38^ (Supplemental Table S1) for phenotypes associated with T2D, lipid metabolism, or inflammation: T2D (PGS005033)^39^, interleukin-1 receptor antagonist protein (IL-1ra, PGS000250)^40^, interleukin-6 (IL-6, PGS000252)^40^, interleukin-8 (IL-8, PGS000254)^40^, tumor necrosis factor receptor 1 (TNF-R1, PGS000287)^40^, C-reactive protein (CRP, PGS002164)^41^, low-density lipoprotein (LDL, PGS004981) cholesterol^39^, high-density lipoprotein (HDL, PGS004086) cholesterol^42^, and triglycerides (TG, PGS002197)^41^. To minimize overfitting, we did not include scores that utilized REGARDS or CNICS in their generation or optimization. Using PLINK, each PRS was calculated for everyone in the training set by multiplying the variant-specific weight by the imputed allelic dosage and summing across the variants. Each PRS was standardized (zero mean, unit standard deviation, SD). We employed elastic net logistic regression^43^ using the R package *glmnet*^44^ to model the association between the PRS and T2D, adjusting for age, age-squared, sex, and 10 PCs. A range of models with different penalties was evaluated using a ten-fold cross-validation. We selected our best model in terms of the highest cross-validated area under the receiving-operating characteristic curve (AUC), incorporating all traits for each metaPRS (inflammation and lipid metabolism). The log odds estimates obtained were used as a constant in the metaPRS models. We generated two metaPRS for each ancestry stratum. One consisted of the weighted average of the standardized scores for T2D and inflammatory markers (meta-inflammation):

$$\:{PRS}_{i}^{meta-inflammation}=\:\frac{{\beta\:}_{1}{Z}_{i1}+\:{\beta\:}_{2}{Z}_{i2}+\:{\beta\:}_{3}{Z}_{i3}+\:{\beta\:}_{4}{Z}_{i4}+\:{\beta\:}_{5}{Z}_{i5}+\:{\beta\:}_{6}{Z}_{i6}}{{\beta\:}_{1}+\:{\beta\:}_{2}+\:{\beta\:}_{3}+\:{\beta\:}_{4}+\:{\beta\:}_{5}+\:{\beta\:}_{6}}$$

where $\:{Z}_{i1},\:{Z}_{i2},\:{Z}_{i3},\:{Z}_{i4},\:{Z}_{i5},\:{Z}_{i6}$ are the zero mean and unit standardized T2D, IL-1ra, IL-6, IL-8, TNF-R1, and CRP risk scores for the *i*th individual, respectively, and $\:{\beta\:}_{1},\:\:{\beta\:}_{2},\:\:{\beta\:}_{3},\:\:{\beta\:}_{4,\:}\:\:{\beta\:}_{5},\:\:{\beta\:}_{6}$ are the coefficient (log odds) for each PRS.

Similarly, the other metaPRS consisted of the weighted average of the standardized scores for lipid metabolism (meta-lipids) plus T2D:

$$\:{PRS}_{i}^{meta-lipids}=\:\frac{{\beta\:}_{1}{Z}_{i1}+\:{\beta\:}_{2}{Z}_{i2}+\:{\beta\:}_{3}{Z}_{i3}+\:{\beta\:}_{4}{Z}_{i4}}{{\beta\:}_{1}+\:{\beta\:}_{2}+\:{\beta\:}_{3}+\:{\beta\:}_{4}}$$

where $\:{Z}_{i1},\:{Z}_{i2},\:{Z}_{i3},\:{Z}_{i4}$ are the zero mean and unit standardized T2D, LDL-C, HDL-C, and TG risk scores for the *i*th individual, respectively, and $\:{\beta\:}_{1},\:\:{\beta\:}_{2},\:\:{\beta\:}_{3},\:\:{\beta\:}_{4\:}$ are the log odds for each PRS.

### Statistical analysis

Baseline characteristics were presented as means and SDs for continuous variables, and as counts and frequencies for categorical data. Each metaPRS developed using the training set was held fixed and evaluated in CNICS (*n* = 4,120 Black; *n* = 3,460 White), as well as the REGARDS validation set (*n* = 2,586 Black; *n* = 1,566 White). The models were stratified by genetic ancestry. Each PRS was evaluated per quintile, as well as per 1-SD. The association of each metaPRS with prevalent T2D was evaluated in CNICS using a multivariable logistic regression model accounting for age, age-squared, sex, first 10 PCs, and genotyping array. A fully adjusted model further accounted for medications including ART and statin use, as well as BMI, SBP, and cigarette smoking status. To assess the predictive utility of these scores for T2D, the AUC was calculated for five models: a base model (Model 1) adjusting for age, age-squared, sex, 10 PCs, and genotyping array; a clinical model (Model 2) adjusting for the base model plus ART use, statin use, BMI, SBP, and cigarette smoking; a clinical model plus T2D PRS model (Model 3), which is the clinical model plus the single trait T2D PRS; a clinical model plus the meta-inflammation PRS (Model 4); and the clinical model plus the meta-lipids PRS (Model 5). Delong’s test for two correlated receiver operator characteristic curves compared the pairwise performance of the models in the R package *pROC*. The fit of each model was determined by Nagelkerke pseudo-R^2^. The net reclassification improvement (NRI) was used to assess the potential for improved discrimination across the models when adding the different PRS. The R package *nricens* was applied for NRI analysis. Additional validation was performed in the holdout subset of REGARDS, with similar models, except for genotyping array, ART use, and statin use. P values are all 2-sided with a 0.05 cutoff for significance. All statistical analyses were conducted using R version 4.3.1 (R Foundation).

## Results

The characteristics of the REGARDS and CNICS participants are shown in Table 1. In the REGARDS training cohort, Black participants (*N* = 6,034) were younger on average (mean age 63.6 ± 9.2 years) than White participants (*N* = 11,972; mean age 64.2 ± 9.1 years). Black participants, compared to White participants, also had a higher average BMI (30.8 kg/m^2^ versus 28.3 kg/m^2^), as well as a higher prevalence of T2D (30.2% versus 14.9%). These trends were also observed in the REGARDS validation cohort. In the CNICS validation cohort, Black participants (*N* = 4,120) were slightly younger (mean age 39.1 ± 10.8 years) and had a higher prevalence of T2D (26.9%) compared to White participants (*N* = 3,460; mean age 40.5 ± 9.9 years; 16.5% T2D).

Using the CNICS validation set, we evaluated the association between the T2D single-trait PRS and our two metaPRS per one SD increase in the PRS, as well as by quintile (Table 2). For Black participants, the single-trait T2D PRS, meta-inflammation PRS, and meta-lipids PRS demonstrated similar patterns of association with T2D. Individuals in the lowest quintile (Q1) had significantly lower odds of T2D compared to the reference group (Q3), with odds ratios (OR) ranging from 0.66 to 0.72 across the three PRS models. Conversely, individuals in the highest PRS quintiles (Q4 and Q5) exhibited increased odds of T2D, particularly in the highest quintile (Q5), with OR = 1.67 (95% CI: 1.33–2.09) for the T2D PRS, OR = 1.45 (95% CI: 1.17–1.82) for the meta-inflammation PRS, and OR = 1.61 (95% CI: 1.29–2.02) for the meta-lipids PRS, indicating a stronger association with increase in quintile. The per-SD increase in PRS was associated with a similar increase in T2D risk across all three models (T2D PRS OR = 1.35, 95% CI: 1.25–1.45; meta-inflammation PRS OR = 1.33, 95% CI: 1.23–1.44; meta-lipids PRS OR = 1.35, 95% CI: 1.25–1.46).

For White participants, the single-trait T2D PRS and both metaPRS followed a similar pattern to Black individuals, with ORs increasing across quintiles, particularly in Q5, where the highest risk estimates were observed (OR = 1.81, 95% CI: 1.37–2.40 for T2D PRS; OR = 1.81, 95% CI: 1.37–2.40 for meta-inflammation PRS; and OR = 1.96, 95% CI: 1.48–2.60 for meta-lipids PRS). The single-trait T2D PRS and the meta-inflammation PRS showed the strongest per-SD effect (OR = 1.57, 95% CI: 1.42–1.73); however, the meta-lipids PRS showed a similarly strong association (OR = 1.53, 95% CI: 1.40–1.69) (Table 2).

Next, we compared the predictive performance of the single-trait T2D PRS and two metaPRS to a base model (Model 1, adjusting for age, age-squared, sex, 10 PCs, and genotyping array). In Black individuals, Model 1 had an AUC of 70.16% (95% CI: 68.36%–71.97%) and a model R² of 14.67%. The addition of clinical risk factors (Model 2, adjusted for Model 1 plus ART use, statin use, BMI, SBP, and cigarette smoking) substantially improved predictive performance compared to Model 1, increasing the AUC to 79.67% (95% CI: 78.16%–81.18%) and the model R² to 30.19%. Furthermore, this model had a net reclassification improvement (NRI) of 16.59% (95% CI: 12.88%–20.80%). Incorporating PRS further improved model performance, with the single-trait T2D PRS (Model 3) yielding the highest AUC (80.41%, 95% CI: 78.91%–81.90%) (Supplemental Figure S1A) and a PRS-specific R² =1.88%. The meta-lipids PRS (Model 5) also contributed meaningfully, achieving the highest NRI of 18.94% (95% CI: 15.08%–22.77%) and PRS R^2^ = 1.91%. However, the improvements from the meta-lipids PRS were modest compared to single-trait PRS (Table 3).

Among White individuals, the overall trends were similar, though AUCs were slightly lower (Table 3). Model 1 had an AUC of 68.70% (95% CI: 66.22%–71.18%) and a model R² of 10.24%, which increased significantly in Model 2 (AUC = 76.51%, 95% CI: 74.34%-78.68%; model R² = 21.31%; NRI = 15.48%, 95% CI: 10.71%-20.36%). The inclusion of PRS provided additional predictive power, with the single-trait T2D PRS (Model 3) showing the strongest improvement (AUC = 78.28%, 95% CI: 76.15%–80.40%; PRS R² = 3.18%) (Supplemental Figure S1B). The meta-inflammation PRS (Model 4) yielded a slightly higher NRI (16.57%, 95% CI: 11.49%–22.17%) compared to the meta-lipid PRS (Model 5). Similar to what we observed in Black CNICS individuals, the most significant improvement in prediction came from traditional clinical risk factors rather than any genetic score alone. To formally evaluate differences in predictive performance, we used DeLong’s test for correlated ROC curves, as summarized in Supplemental Table S2. When incorporating clinical risk factors (Model 2) and genetic information (Models 3–5), predictive performance improved significantly (*p* < 0.05) compared to the base model (Model 1). The addition of PRS models further enhanced the prediction compared to clinical risk factors alone, both examining the PRS per quintile, as well as per SD; however, comparisons between different PRS models were not significant.

We were also interested in validating the performance in a subset of non-overlapping REGARDS participants. In REGARDS Black participants, those in the highest quintile (Q5) had significantly higher odds of T2D compared to the reference group (Q3) for all PRS models, with a 2-fold increase in the odds using the meta-lipids PRS (OR = 2.06, 95% CI:1.57–2.70). Similarly, all participants in Q1 had a reduction of odds of T2D compared to Q3, ranging from 38% to 45%. A 1-SD increase in PRS was associated with 62–63% higher odds of T2D (Supplemental Table S3). Among White individuals, the effect sizes were more pronounced. The highest quintile (Q5) showed a substantial increase in T2D risk compared to Q3, with odds ratios ranging from 2.28 (95% CI: 1.57–3.32) for the meta-inflammation PRS to 2.75 (95% CI: 1.89–4.04) for the meta-lipids PRS. A 1-SD increase was associated with over a 2-fold increase in odds for all PRS scores (Supplemental Table S3).

Supplemental Table S4 presents prediction results from the REGARDS validation cohort. Among Black participants, the base model (Model 1) had a modest AUC of 57.89% (95% CI: 55.44% − 60.34%) and a full model R^2^ of 2.23%. The addition of clinical risk factors in Model 2 increased the AUC to 65.25% (95% CI: 62.98, 67.59%) and the R^2^ to 8.65%, with an NRI of 8.43% (95% CI: 3.29%-13.42%). Including the single-trait T2D PRS (Model 3) further improved prediction (AUC = 69.60%, 95% CI: 67.36%-71.84%; R^2^ = 13.84%; NRI = 17.31%, 95% CI: 11.95%-22.94%) (Supplemental Figure S2A). Similar model enhancements were found in Models 4 and 5, with the highest AUC, model R^2^, and NRI observed for the meta-inflammation PRS (AUC = 69.68%, 95% CI: 67.44–71.92%; R^2^ = 14.03%; NRI = 18.15%, 95% CI: 12.88%-23.28%). In White participants, the predictive performance was higher, with the Model 1 AUC = 58.52% (54.96%-62.07%) and R^2^ = 2.23%. The AUC increased to 76.77% (73.74%-79.79%) and an R^2^ = 22.59% in Model 5, with the addition of the meta-lipids PRS (Supplemental Figure S2B). Similar to the CNICS results, the REGARDS validation cohort shows significant improvements in predictive performance when clinical risk factors (Model 2) and genetic information via PRS per SD (Models 3–5) are added to Model 1. The most significant improvements are between Models 1 and 4 for Black (*p* = 5.62E-18) participants and Models 1 and 5 for White participants (*p* = 3.61E-20) participants (Supplemental Table S2).

## Discussion

Transferability across populations both ancestral and clinical remains a key priority in the development of PRS. Several PRS have been developed for T2D, and multi-ancestry versions derived from large summary datasets have promise for predicting disease in diverse populations. Further, meta scores, which combine PRS for the trait of interest and its risk factors, have demonstrated improvements over single trait scores for other chronic diseases, such as stroke^17^ and CAD^18^. In an analysis of 12,740 self-reported Black (~ 32% with HIV) and 5,063 self-reported White (~ 68% with HIV) participants from CNICS and the REGARDS study, we evaluated the utility of metaPRS models for predicting T2D in populations with and without HIV across two ancestry groups, using existing genetic and phenotypic data. Our results confirm the transferability of single-trait PRS across ancestries and HIV status, but metaPRS showed no meaningful improvement regarding prediction accuracy, suggesting that combining lipid/inflammation PRS adds limited value in these populations. Our results highlight the transferability of a well-validated PRS, but our metaPRS trained for inflammation and lipid metabolisms show no meaningful improvement in prediction for T2D.

To our knowledge, few prior studies have applied a meta-scoring approach to T2D risk prediction using genetic data for risk factor traits. One such study integrated variants across T2D-related traits and demonstrated improved discrimination and reclassification over conventional methods^45^. While that work established the utility of trait-informed meta-genomic risk scores for T2D, our study builds on this by incorporating trait-specific scores for lipids and inflammation and evaluating performance in two demographically distinct cohorts. We found that genetic risk prediction models incorporating the metaPRS did not improve risk discrimination compared to single-trait PRS for T2D according to the AUC and NRI (models 3–5 in Table 3 and Supplemental Table 4). Consistent with other chronic diseases, we found demographic factors and traditional clinical risk factors were most important for T2D risk prediction in both CNICS and REGARDS. Notably, AUCs were slightly higher in CNICS than in REGARDS, which could be due to the younger age of the CNICS cohort compared to the REGARDS cohort and additional clinical risk factor data in CNICS, including ART. Overall, the added benefit of PRS above clinical risk factors in our risk prediction models was moderate (PRS R^2^ ranging ~ 2%-10%), but on par with other studies^14,46–48^. As PRS methods improve and other biomarker scores become available, we expect metaPRS to improve for T2D in the general population and PWH.

Our findings align with previous studies evaluating the performance of single-trait PRS derived from multi-ancestry data for T2D in both Black and White individuals. In a prior CNICS study, Cheng et al. developed a multiethnic PRS and reported that individuals in the top 5% of the distribution had 2-fold increased odds of T2D in White participants and 59% increased odds of T2D in Black participants^32^. Similarly, in general population studies, Ge et al. found that individuals had a ~ 1.5 to 2-fold increased odds of T2D per 1 SD of the PRS across Black and White participants, respectively, with liability R^2^ estimates ranging from 2.8% (Black) to 9.2% (White) in these individuals^14^.

While PRS derived from the general population retain predictive utility in PWH, their effects are modest relative to clinical factors and may be diminished by HIV-specific pathways (e.g., ART-related metabolic changes). For example, cardiometabolic PRS have been associated with subclinical CAD in PWH, particularly with severe phenotypes such as stenosis > 50% and non-calcified vulnerable plaque, independent of demographic and HIV-related factors^49^. In a separate HIV cohort, a CAD PRS comprising 23 SNPs identified in the general population was independently associated with CAD events and notably explained more variance than traditional clinical risk factors like T2D or hypertension^50^. Prior work in CNICS has shown that PRS for lipid traits, T2D, and myocardial infarction, using summary statistics from the general population, are significantly correlated with phenotypes in PWH, suggesting that genetic contributions remain informative in the context of HIV. The same study also reported genome-wide associations for cardiometabolic traits among PWH^32^. Building on this foundational work, our study tests the transferability of PRS derived from large-scale, general population studies and provides complementary insight into the potential to inform T2D risk prediction in PWH.

Although we observed smaller effect sizes and R^2^ values in CNICS compared to REGARDS, this may reflect differential ascertainment, clinical context, or interactions with HIV-related exposures^51^. In the absence of large HIV-specific T2D GWAS datasets, our findings suggest that multi-ancestry single-trait PRS for T2D, developed in the general population, remain informative for both Black and White PWH, although their predictive utility may be reduced due to HIV-related factors. Notably, metaPRS did not outperform single-trait PRS in either cohort, possibly because the lipid and inflammation scores did not provide independent predictive signals beyond those captured by the T2D PRS. Future studies should aim to develop and validate HIV-specific PRS for T2D to improve risk prediction in this population.

The strengths of our study include the assessment of PRS transferability across ancestries and HIV status in the largest cohort of PWH with genetic data to date, which contributes to the growing body of literature on genetic prediction in diverse populations^52,53^. Our study is unique in evaluating metaPRS performance in a high-risk population of PWH and across ancestries, providing insight into the utility of genetic risk prediction tools in underrepresented groups for cardiometabolic diseases, including T2D.

However, this study is not without limitations. First, PRS used to create the metaPRS were primarily derived from European populations, limiting generalizability to non-European ancestries. Larger studies in diverse populations are needed to enhance PRS accuracy and applicability. Second, the PRS constructed were from HIV-negative cohorts, however, we note that HIV status may have been missed or not recorded in those cohorts. Importantly, as more GWAS data becomes publicly available, future T2D PRS work in HIV populations should consider PRS trained in HIV-specific GWAS data for comparison to general population scores. Third, we were limited by availability of biologically relevant PRS for metaPRS construction by those traits published in the PGS catalog. Fourth, the inclusion of multiple PRS methodologies may have introduced additional variability, although we standardized scores to mitigate this effect. Fifth, we were unable to fully account for lifestyle and environmental factors, such as diet and physical activity, which may influence the observed associations and contribute to residual confounding. Sixth, HIV-related chronic inflammation, which is distinct from inherited inflammatory pathways, may obscure genetic effects, thereby diminishing the contribution of inflammation-related variants to T2D risk in PWH. Seventh, definitions of T2D differed slightly between REGARDS and CNICS, with REGARDS relying primarily on glucose measures and medication use, while CNICS used hemoglobin A1c, medication data, and diagnostic codes. These differences may have contributed to some degree of misclassification and variability in predictive performance across cohorts. Finally, our analysis was limited to prevalent T2D cases, potentially leading to misclassification and limiting generalizability to incident T2D.

In summary, while metaPRS showed no meaningful improvement in T2D prediction, single-trait PRS and traditional clinical risk factors remain critical components of T2D risk prediction. Future studies should focus on refining PRS models using larger, diverse populations and exploring the contributions of HIV-specific genetic factors to T2D risk in PWH. Leveraging genetic biomarkers, such as PRS and metaPRS, can serve as a tool for individualized prevention and management in precision medicine.

**Table 1:** Demographic and clinical characteristics of training and validation cohorts.

	Training		Validation			
	REGARDS		CNICS		REGARDS	
	Black	White	Black	White	Black	White
N	6,034	11,972	4,120	3,460	2,586	1,566
Age (years), mean (SD)	63.6 (9.2)	64.2 (9.1)	39.1 (10.8)	40.5 (9.9)	63.6 (9.1)	68.4 (10.4)
Sex, N (%)						
Male	2,396 (39.7)	5,844 (48.8)	2,805 (68.1)	3,090 (89.3)	981 (37.9)	916 (58.5)
Female	3,638 (60.3)	6,121 (51.2)	1,315 (31.9)	370 (10.7)	1,605 (62.1)	650 (41.5)
T2D, N (%)	1,789 (30.2)	1,781 (14.9)	1,109 (26.9)	571 (16.5)	716 (28.1)	303 (19.3)
BMI (kg/m^**2**^**),** mean (SD)	30.8 (6.8)	28.3 (5.6)	28.4 (7.5)	27.0 (5.7)	30.9 (6.7)	28.3 (5.4)
Current Smoking, N (%)	1,084 (18.0)	1,581 (13.2)	1,166 (28.3)	1,004 (35.5)	463 (18.0)	231 (14.8)
SBP, mean (SD)	131.1 (17.5)	124.9 (15.5)	129.0 (11.4)	125.7 (9.8)	130.9 (17.2)	128.1 (16.9)
Presence of ART, N (%)	-	-	3,967 (96.3)	3,319 (95.9)	-	-
Presence of statin use, N (%)	-	-	1,922 (46.7)	1,696 (49.0)	-	-

SD = standard deviation; T2D = type 2 diabetes; BMI = body mass index; SBP = systolic blood pressure; ART = antiretroviral therapy.

**Table 2:** Odds ratios associated with T2D for single-trait and metaprs among CNICS validation set.

Ancestry	PRS Unit	Odds Ratio (95% CI)		
		Single Trait T2D PRS	Meta-Inflammation PRS	Meta-Lipids PRS
AFR	Q1	0.68 (0.53, 0.87)	0.66 (0.51, 0.84)	0.72 (0.56, 0.93)
	Q2	1.03 (0.81, 1.30)	0.72 (0.57, 0.91)	0.95 (0.75, 1.20)
	Q3	Ref.	Ref.	Ref.
	Q4	1.17 (0.92, 1.47)	0.89 (0.71, 1.12)	1.19 (0.95, 1.50)
	Q5	1.67 (1.33, 2.09)	1.45 (1.17, 1.82)	1.61 (1.29, 2.02)
	Per 1 SD increase	1.35 (1.25, 1.45)	1.33 (1.23, 1.44)	1.35 (1.25, 1.46)
EUR	Q1	0.53 (0.37, 0.73)	0.53 (0.38, 0.74)	0.61 (0.43, 0.85)
	Q2	0.64 (0.46, 0.89)	0.65 (0.47, 0.90)	0.84 (0.61, 1.15)
	Q3	Ref.	Ref.	Ref.
	Q4	1.37 (1.03, 1.83)	1.32 (0.99, 1.76)	1.49 (1.11, 1.99)
	Q5	1.81 (1.37, 2.40)	1.81 (1.37, 2.40)	1.96 (1.48, 2.60)
	Per 1 SD increase	1.57 (1.42, 1.73)	1.57 (1.42, 1.73)	1.53 (1.40, 1.69)

Adjusted for age, age squared, sex, ten PCs, and genotyping array.

T2D = type 2 diabetes; PRS = polygenic risk score; AFR = African ancestry; EUR = European ancestry; SD = standard deviation.

**Table 3:** Prediction results from CNICS validation set.

Ancestry	Model	AUC (95% CI) in %	Full Model *R*^2^ in %	PRS *R*^2^ in %	NRI (95% CI) in %
AFR	Model 1	70.16 (68.36, 71.97)	14.67	-	Ref.
	Model 2	79.67 (78.16, 81.18)	30.19	-	16.59 (12.88, 20.80)
	Model 3	80.41 (78.91, 81.9)	31.80	1.88	18.59 (15.27, 22.09)
	Model 4	80.31 (78.81, 81.81)	31.66	1.75	18.31 (14.67, 21.98)
	Model 5	80.44 (78.94, 81.93)	31.83	1.91	18.94 (15.08, 22.77)
EUR	Model 1	68.7 (66.22, 71.18)	10.24	-	Ref.
	Model 2	76.51 (74.34, 78.68)	21.31	-	15.48 (10.47, 20.51)
	Model 3	78.28 (76.15, 80.4)	24.44	3.18	17.09 (11.72, 22.60)
	Model 4	78.32 (76.20, 80.44)	24.48	3.20	16.57 (11.49, 22.17)
	Model 5	78.11 (75.98, 80.24)	24.15	2.87	15.72 (10.07, 21.17)

Model 1 adjusts for age, age squared, sex, 10 PCs, and genotyping array.

Model 2 adjusts for Model 1 plus ART use, statin use, BMI, SBP, and cigarette smoking.

Model 3 adjusts for Model 2 plus single-trait T2D PRS per SD.

Model 4 adjusts for Model 2 plus meta-inflammation PRS per SD.

Model 5 adjusts for Model 2 plus meta-lipids PRS per SD.

AUC = area under the ROC curve; PRS = polygenic risk score; AFR = African ancestry; EUR = European ancestry; NRI = net reclassification improvement; ART = antiretroviral therapy; BMI = body mass index; SBP = systolic blood pressure

## Supplementary Information

Below is the link to the electronic supplementary material.

Supplementary Material 1

Supplementary Material 2

## Data Availability

The REGARDS (dbGaP accession: phs002719.v1.p1) and CNICS (dbGaP accession: phs001788.v1.p1) phenotypic and genetic data are available through the database of Genotypes and Phenotypes (dbGaP; https://www.ncbi.nlm.nih.gov/gap/) under controlled access. In addition, REGARDS data supported by the National Institute on Aging (NIA) and the National Institute of Neurological Disorders and Stroke (NINDS) through the Center for Alzheimer’s and Related Dementias (CARD) will be available via the AD Knowledge Portal (https://adknowledgeportal.synapse.org).
