# Supplementary material for: Genomic risk prediction of type 2 diabetes in people living with and without HIV
Source: Sci Rep. 2026 Jan 22;16:3078. doi: 10.1038/s41598-025-31471-7 (PMC12830814; doi:10.1038/s41598-025-31471-7)
Supplement: Supplementary file 2 — Supplementary Material 2 [file 41598_2025_31471_MOESM2_ESM.docx]

***Supplemental Material***

**Genomic risk prediction of Type 2 Diabetes in people living with and without HIV**

Nicole D. Armstrong^1*^, Vinodh Srinivasasainagendra^2^, Lavanya Pilla^2^, Radhika Gangaraju^3^, Peter W. Hunt^4^, Robin M. Nance^5^, Heidi M. Crane^5^, Inga Peter^6^, Sonya L. Heath^3^, Greer A. Burkholder^3^, Richard D. Moore^7^, Jeffrey M. Jacobson^8^, Edward R. Cachay^9^, Thibaut Davy-Mendez^10, 11^, Hirotaka Iwaki^12,13^, Lana Sargent^12,14^, Hemant K. Tiwari^2^, Marguerite R. Irvin^1^

^1^ Department of Epidemiology, University of Alabama at Birmingham, Birmingham, AL, USA

^2^ Department of Biostatistics, University of Alabama at Birmingham, Birmingham, AL, USA

^3^ Department of Medicine, University of Alabama at Birmingham, Birmingham, AL, USA

^4^ Division of Experimental Medicine, University of California, San Francisco, San Francisco, CA, USA

^5^ Department of Medicine, University of Washington, Seattle, WA, USA

^6^ Department of Genetics and Genomic Sciences, Icahn School of Medicine at Mount Sinai, New York, NY, USA

^7^Department of Medicine, Johns Hopkins University, Baltimore, MD, USA

^8^Department of Medicine, Case Western Reserve University, Cleveland, OH, USA

^9^Department of Medicine, Division of Infectious Diseases, Mayo Clinic, Scottsdale, Arizona, USA,

^10^School of Medicine, University of North Carolina at Chapel Hill, Chapel Hill, NC

^11^Gillings School of Public Health, University of North Carolina at Chapel Hill, Chapel Hill, NC, USA

^12^Center for Alzheimer’s and Related Dementias, National Institute on Aging, Bethesda, MD, USA

^13^School of Nursing, Virginia Commonwealth University, Richmond, VA, USA

*** Correspondence:**Corresponding Author: Nicole D. Armstrong
nmda@uab.edu

**Keywords:** polygenic risk score, precision medicine, type 2 diabetes, HIV


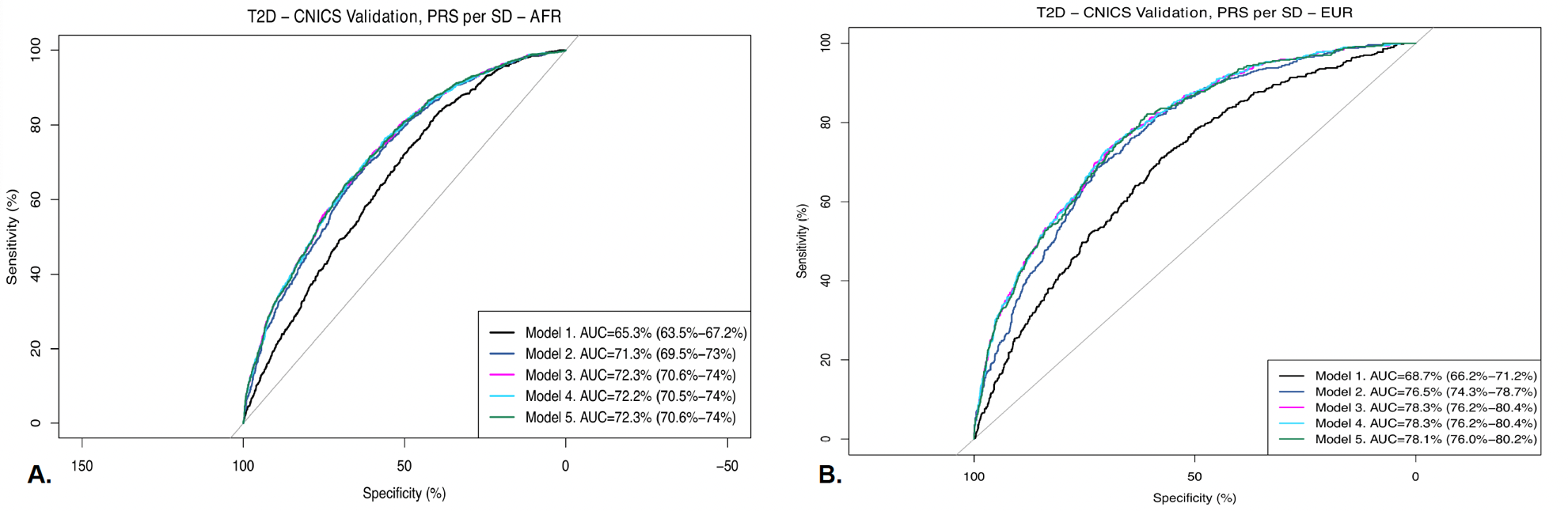


**Supplemental Figure 1. Area under the ROC for prediction of T2D using PRS models per standard deviation (SD) in CNICS (A) Black participants and (B) White participants.** Model 1 adjusts for age, age-squared, sex, first 10 PCs, and genotyping array. Model 2 adjusts for Model 1 plus BMI, SBP, cigarette smoking, ART use, and statin use. Model 3 adjusts for Model 2 plus the single-trait T2D PRS. Model 4 adjusts for Model 2 plus the meta-inflammation PRS. Model 5 adjusts for Model 2 plus the meta-lipids PRS.


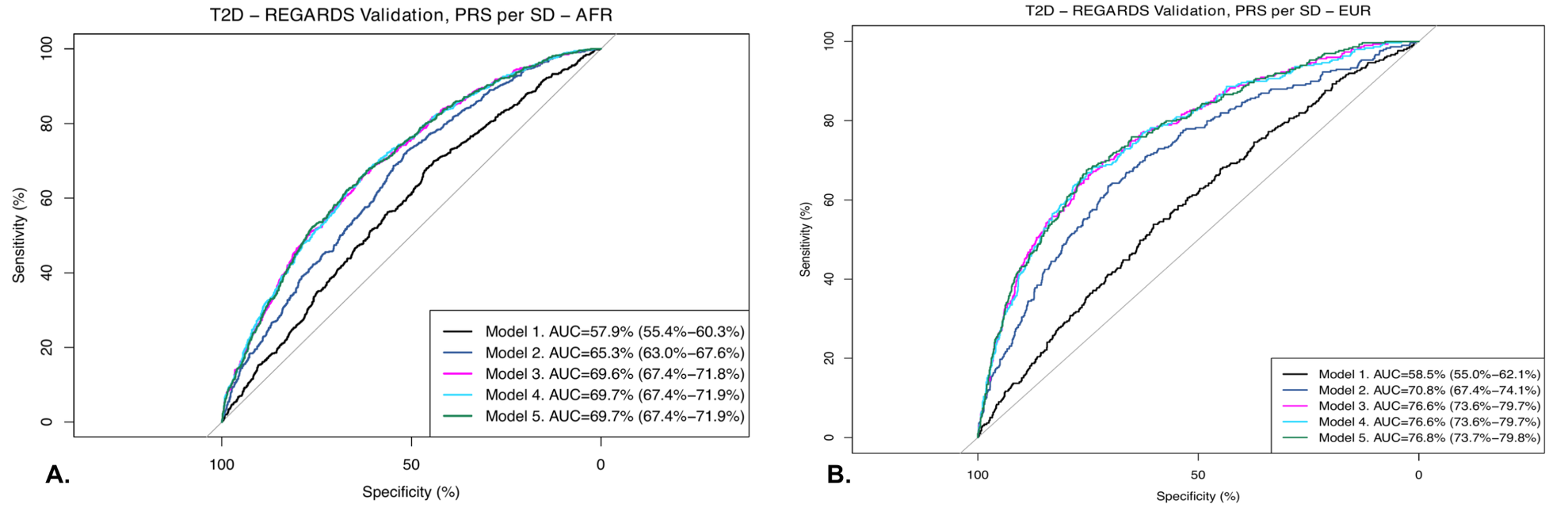


**Supplemental Figure 2. Area under the ROC for prediction of T2D using PRS models per standard deviation (SD) in REGARDS (A) Black participants and (B) White participants.** Model 1 adjusts for age, age-squared, sex, and first 10 PCs. Model 2 adjusts for Model 1 plus BMI, SBP, and cigarette smoking. Model 3 adjusts for Model 2 plus the single-trait T2D PRS. Model 4 adjusts for Model 2 plus the meta-inflammation PRS. Model 5 adjusts for Model 2 plus the meta-lipids PRS.
